# Integrin β6 deficiency protects mice from experimental colitis and colitis-associated carcinoma by altering macrophage polarization

**DOI:** 10.3389/fonc.2023.1190229

**Published:** 2023-05-08

**Authors:** Qi Sun, Zhihua Lu, Lei Ma, Dong Xue, Chang Liu, Changchun Ye, Wenbo Huang, Yueyan Dang, Fanni Li

**Affiliations:** ^1^ Department of General Surgery, The First Affiliated Hospital of Xi’an Jiaotong University, Xi’an, China; ^2^ Department of General Surgery, Qilu Hospital (Qingdao), Cheeloo College of Medicine, Shandong University, Qingdao, China; ^3^ Department of Anesthesiology and Perioperative Medicine, The First Affiliated Hospital of Xi’an Jiaotong University, Xi’an, China; ^4^ Department of Talent Highland, The First Affiliated Hospital of Xi’an Jiaotong University, Xi’an, China

**Keywords:** integrin αvβ6, inflammatory bowel disease, colitis-associated carcinoma, macrophage polarization, dextran sodium sulfate

## Abstract

**Background:**

Given the key role of integrins in maintaining intestinal homeostasis, anti-integrin biologics in inflammatory bowel disease (IBD) are being investigated in full swing. However, the unsatisfactory efficacy and safety of current anti-integrin biologics in clinical trials limit their widespread use in clinic. Therefore, it is particularly important to find a target that is highly and specifically expressed in the intestinal epithelium of patients with IBD.

**Methods:**

The function of integrin αvβ6 in IBD and colitis-associated carcinoma (CAC) with the underlying mechanisms has been less studied. In the present study, we detected the level of integrin β6 within inflammation including colitis tissues in human and mouse. To investigate the role of integrin β6 in IBD and CAC, integrin β6 deficient mice were hence generated based on the construction of colitis and CAC model.

**Results:**

We noted that integrin β6 was significantly upregulated in inflammatory epithelium of patients with IBD. Integrin β6 deletion not only reduced infiltration of pro-inflammatory cytokines, but also attenuated disruption of tight junctions between colonic epithelial cells. Meanwhile, lack of integrin β6 affected macrophage infiltration in mice with colitis. This study further revealed that lack of integrin β6 could inhibit tumorigenesis and tumor progression in CAC model by influencing macrophage polarization, which was also involved in attenuating the degree of intestinal symptoms and inflammatory responses in mice suffering from colitis.

**Conclusions:**

The present research provides a potentially new perspective and option for the treatment of IBD and CAC.

## 1 Introduction

Inflammatory bowel disease (IBD) is a group of immunity-mediated chronic recurrent inflammatory diseases of the gastrointestinal tract. Patients with IBD mainly present with symptoms such as abdominal pain, diarrhea, blood in the stool and weight loss (1). Although the etiology of IBD remains obscure, innate genetic susceptibility and external environment aspects are thought to contribute to immune dysregulation, disruption of the intestinal barrier and loss of tolerance to intestinal commensal bacteria (2). Long-term recurrent chronic inflammation can trigger oncogenic injury of colonic epithelial cells, leading to tumor initiation and development. Patients suffering from IBD, mainly including Crohn’s disease (CD) and ulcerative colitis (UC), have an increased risk of colitis-associated carcinoma (CAC) (3). CD resulted in an 8% increase in the cumulative risk of developing CAC after 30 years of disease, while UC increased the risk by 18-20% (4). As the worldwide incidence continues to rise, IBD poses a huge health and economic burden on society.

Current pharmacological management of IBD is primarily based on the use of corticosteroids, 5-aminosalicylates and biologic agents aimed at relieving intestinal inflammation and controlling clinical symptoms. Despite of the wide range of available drugs, there is still a high percentage of patients demonstrating initial no-response, loss of response, relapse or adverse reactions, thus requiring extra therapeutic solutions (5). The therapeutic difficulty and carcinogenic risk of IBD render it particularly significant to identify novel potential targets.

Integrins are heterodimeric glycoproteins that span membranes and occupy a pivotal position among receptors engaged in cell adhesion. By binding to the extracellular matrix (ECM), integrins trigger intracellular signaling that regulates a wide variety of cellular behaviors including survival, proliferation, migration, tissue invasion, intrinsic immunity and diverse cell destiny transitions (6). Integrins are composed of α and β subunits through non-covalent binding. There are 18 α subunits and 8 β subunits in mammalian cells, giving rise to 24 different receptor isoforms, each with specific recognition ligands and unique tissue distributions (7). Integrin β6 can only form heterodimeric receptor complex with integrin αv subunit, therefore the expression of integrin αvβ6 is determined by the gene *ITGB6* encoding integrin β6, which is expressed only in epithelial cells (8).

Although upregulation of integrin αvβ6 has been shown to occur during development, wound healing, fibrosis and tumorigenesis, all of which require tissue remodeling (9), the role of integrin αvβ6 in IBD and CAC has been rarely studied. In the present study we examined the level of integrin β6 in inflammation including colitis tissues. Integrin β6 deficient (*ITGB6*-knockout) and wild-type (WT) mice were subsequently used to construct dextran sodium sulfate (DSS)-induced acute colitis mouse model and azoxymethane (AOM)/DSS-induced CAC mouse model to explore the role of integrin β6 in IBD and CAC. This study confirmed that integrin β6 deletion affected macrophage infiltration and polarization, thereby attenuating the degree of intestinal inflammatory response and symptoms, and inhibiting tumorigenesis and tumor progression. Our study provides a new strategy and perspective on the therapeutic aspects of IBD and CAC.

## 2 Materials and methods

### 2.1 Immunohistochemistry (IHC)

Paraffin-embedded sections of formalin-fixed tissue specimens were degreased with xylene and rehydrated using a gradient series of alcohols. Heat-induced antigen retrieval was performed after blocking with PBS containing 10% fetal bovine serum. The sections were then treated by incubation with primary and secondary antibodies. Immunohistochemical staining was performed with 3,3’-diaminobenzidine. Primary antibodies were as follows: anti-integrin αvβ6 antibody (Cell Signaling Technology or Millipore Sigma), anti-IL-6 antibody (Abcam), anti-IL-1β antibody (Abcam), anti-TNF-α antibody (Abcam), anti-COX-2 antibody (Abcam) and anti-Ki-67 antibody (Abcam). For the assessment of immunostaining, the immunoreactivity score was determined by multiplying the percentage of positively stained cells and the staining intensity (ranging from 0 to 12). The percentage of positivity was ranked as follows: 0, 0%; 1, 1-10%; 2, 11-50%; 3, 51-75%; 4, >75%. Staining intensity was graded as follows: 0, none; 1, weak; 2, moderate; 3, strong.

### 2.2 Quantitative real-time PCR (qPCR)

Total RNA was isolated from human and mouse colon tissue to be tested using TRIzol reagent (Invitrogen) following the manufacturer’s instructions. After quantification, total RNA was reverse transcribed to cDNA using the PrimeScript RT kit (Takara). qPCR was performed using Thunderbird SYBR Master Mix (Takara) according to the manufacturer’s instructions. The following primers were used: *ITGB6*, forward 5′- ATGGGGATTGAGCTGGTCTG-3′ and reverse 5′- GACAGGTGGGTGAAATTCTCC-3′; *GAPDH*, forward 5′- AGGTCGGTGTGAACGGATTTG -3′ and reverse 5′- GGGGTCGTTGATGGCAACA -3′. *GAPDH* was used as an internal reference. The 2^-ΔΔCt^ method was used and statistical analysis was performed using the Student’s t-test.

### 2.3 Experimental mice

*ITGB6*-knockout (β6-KO) and Wild-type (WT) mice were generated and purchased from the Model Animal Research Center of Nanjing University. All the 8-10 weeks old male mice used in the experiments were reared under specific pathogen-free conditions. The above experiments were approved by the Ethics Committee of The First Affiliated Hospital of Xi’an Jiaotong University.

### 2.4 Mouse model of DSS-induced colitis

Acute colitis was induced in WT and ITGB6-KO mice by feeding drinking water dissolved with a concentration of 2.5% DSS (MP Biomedicals) for 7 days. Body weight, fecal concentration and rectal bleeding were recorded once a day which constitute the disease activity index (DAI) (10). The DAI score was obtained by summing the scores for weight loss, fecal concentration and degree of blood in the stool (range 0-12). On day 7, the length of colon was measured.

### 2.5 Histological assessment

For histological analysis, the inflamed colon was dissected and fixated in formalin solution, which was then paraffin-embedded and sliced into 4 μm-thick slices. Finally, the slices were stained using hematoxylin-eosin (HE) staining kit and alcian blue periodic acid schiff (AB-PAS) staining kit (Abcam). The severity of inflammation was assessed using histology scores as described before (11).

### 2.6 ELISA assay for pro-inflammatory cytokines

To detect the level of pro-inflammatory cytokines (IL-6, TNF-α and IL-1β) in mouse colon tissues, the collected tissue specimens were rinsed in ice-cold saline to remove blood and then grinded into 10% tissue homogenates, which were centrifuged at 3000 rpm at 4°C for 15 min. The supernatant was collected and detected by ELISA according to the instructions using an MCYTOMAG-70K Kit (Merck).

### 2.7 Immunofluorescence assay

Frozen sections of mice colon tissues were first fixed in acetone and permeabilized, and then blocked with 5% normal goat serum at room temperature for 1 h before incubating with primary antibody followed by secondary antibody. Nuclei were stained with 4’,6-diamidino-2-phenylindole (DAPI) working solution. The fluorescent staining of colonic tissue was observed using a fluorescent microscope (Nikon) and images were obtained using Case Viewer software (version 2.3, 3D Histech). The average intensity of immunofluorescence staining was analyzed semi-quantitatively using ImageJ software. The primary antibodies were used as follows: anti-ZO-1 antibody (SantaCruz Biotechnology), anti-occludin antibody (SantaCruz Biotechnology), anti-F4/80 antibody (Cell Signaling Technology), anti-CD86 antibody (Cell Signaling Technology), anti-CD206 antibody (Cell Signaling Technology) and anti-integrin β6 antibody (Cell Signaling Technology).

### 2.8 Mouse model of CAC

Both *ITGB6*-KO mice and wild-type mice were injected intraperitoneally with 10 mg/kg AOM (MedChemExpres) once on day 1. After that, the mice were given normal drinking water for one week, followed by 2.5% DSS drinking water for one week, and then replaced with normal drinking water for two weeks as one cycle. After 3 consecutive cycles, on day 100 after AOM injection, the colon specimens were obtained for subsequent analysis.

### 2.9 Statistical analysis

Statistical analysis was performed and plotted using GraphPad Prism 7 software. Differences between the two groups were compared using a two-tailed Student’s t-test or Mann-Whitney U test analysis. Other data were analyzed using one-way ANOVA followed by Tukey’s test. *P* < 0.05 indicated statistical significance.

## 3 Results

### 3.1 Integrin β6 was upregulated in inflammatory tissues of IBD and associated with disease activity

**Figure 1:**
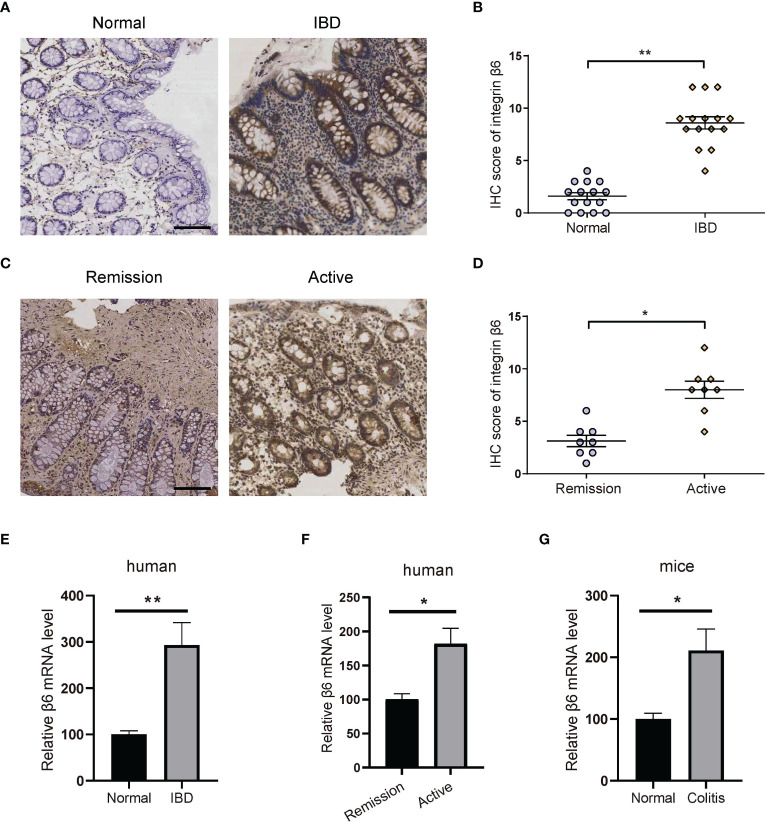
Integrin β6 expression became elevated in IBD and was associated with inflammation severity. **(A)** Representative IHC staining of integrin β6 and **(B)** analysis of IHC score in normal and inflamed colonic tissues obtained from patients with IBD. **(C)** Representative IHC staining of integrin β6 and **(D)** analysis of IHC score in affected colonic tissues at active or remission phase from patients with IBD. **(E)** The mRNA level of integrin β6 in affected colonic tissue obtained from patients with IBD and matched normal colonic tissue. **(F)** The mRNA level of integrin β6 in affected colonic tissues from patients with IBD at active and remission phase. **(G)** The mRNA level of integrin β6 in affected colonic tissues of mice with DSS-induced colitis and matched adjacent normal intestinal tissues. Data represented the means ± SEM, ^*^ *p* < 0.05, ^**^ *p* < 0.01. Scale bar, 100 μm. n = 3 independent experiments.

To investigate the relationship between integrin β6 expression and IBD, we firstly examined the expression of integrin β6 in the inflamed colon of patients with IBD versus normal colon tissues through immunohistochemistry (IHC). Integrin β6 is barely detected in normal colonic epithelium but became overexpressed in IBD tissues (**Figures 1A, B**). Moreover, the expression of integrin β6 was higher in the colonic epithelium of patients with active IBD than those at remission phase (**Figures 1C, D**). Accordingly, qPCR assay also confirmed the increased mRNA level of integrin β6 in affected colonic epithelium of patients with IBD, especially at active phase (**Figures 1E, F**). Likewise, mRNA expression of integrin β6 became significantly elevated in DSS-induced colitis mouse model (**Figure 1G**), indicating that integrin β6 became upregulated in IBD and was associated with disease severity.

### 3.2 Integrin β6 became upregulated during inflammation process

**Figure 2:**
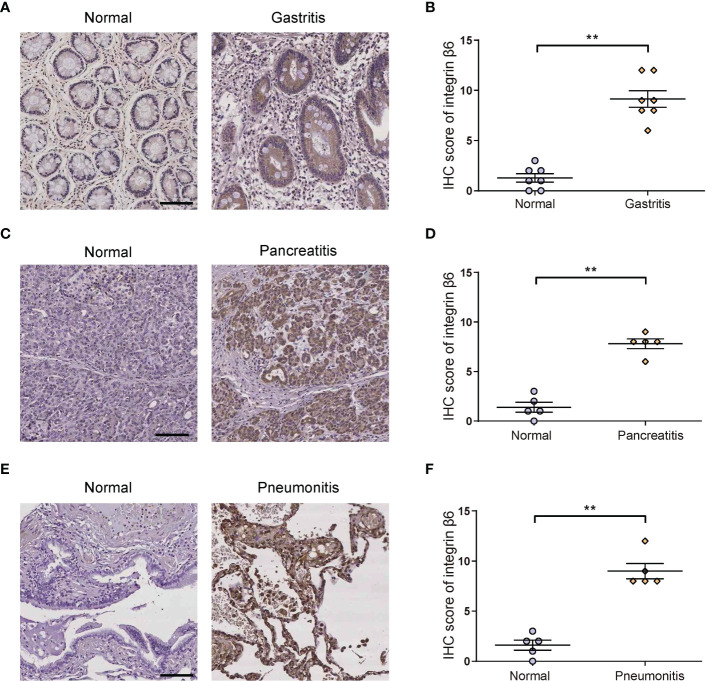
The expression of integrin β6 was increased in human gastritis, pancreatitis and pneumonitis tissues. **(A)** Representative IHC staining of integrin β6 and **(B)** analysis of IHC score in affected gastric mucosal tissue and matched normal gastric mucosa. **(C)** Representative IHC staining of integrin β6 and **(D)** analysis of IHC score in affected pancreatic tissue and matched normal pancreatic tissue. **(E)** Representative IHC staining of integrin β6 and **(F)** analysis of IHC score in affected pneumonia tissues and matched normal lung tissue. Data represented the means ± SEM, ^**^ *p* < 0.01. Scale bar, 100 μm.

To verify the relationship between integrin β6 and inflammation, we further examined human gastritis, pancreatitis and pneumonia tissue specimens with corresponding normal tissues, respectively. It was found that integrin β6 expression was significantly elevated in gastritis (**Figures 2A, B**), pancreatitis (**Figures 2C, D**) and pneumonitis (**Figures 2E, F**). These findings suggested that the upregulation of integrin β6 was closely related to inflammation.

### 3.3 Integrin β6 deficient mice were resistant to DSS-induced colitis

**Figure 3:**
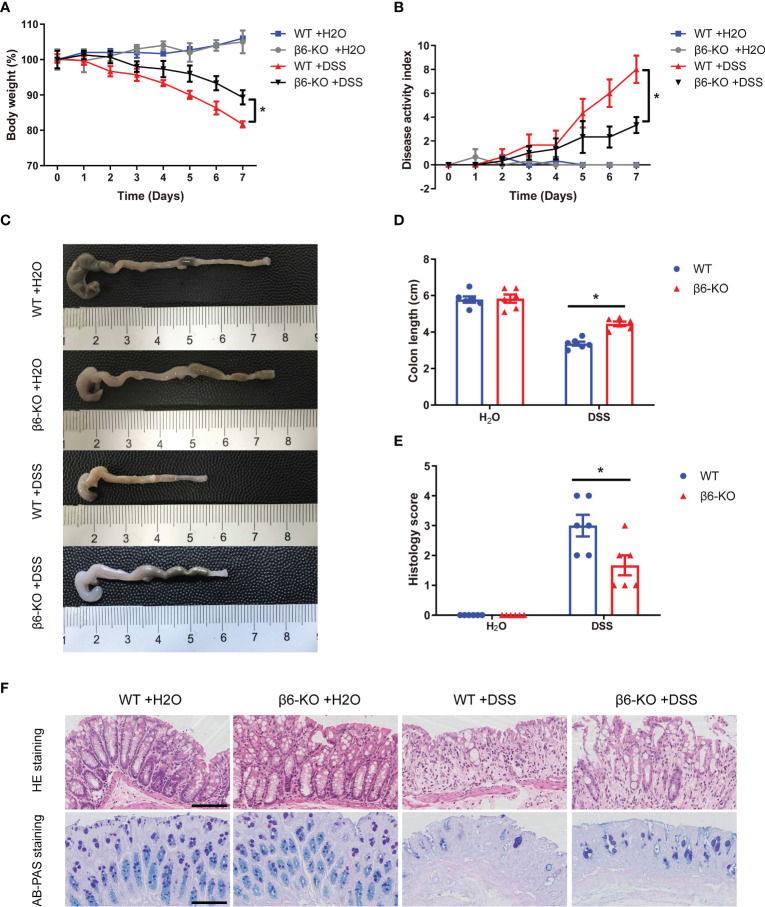
*ITGB6* knockout attenuated the susceptibility of mice to DSS-induced acute colitis. **(A-F)** WT and integrin β6-KO mice were treated in the presence or absence of 2.5% DSS for 7 days, and main symptoms of IBD were evaluated. **(A)** Body weight change; **(B)** DAI score; **(C)** Representative colon images and **(D)** quantitative measurement of colon length; **(E)** Histological score of disease activity; and **(F)** Representative images of HE staining and AB-PAS staining of colonic mucosa. Data represented the means ± SEM, ^*^ *p* < 0.05. Scale bar, 50 μm.

In order to investigate the role of integrin β6 in IBD, *ITGB6*-knockout (β6-KO) and wild-type (WT) mice model of acute colitis was constructed by the presence of 2.5% DSS solution. On day 7 after DSS administration, the body weights of β6-KO mice were heavier, with a lower disease activity index (DAI), than those of WT mice (**Figures 3A, B**). Meanwhile, there was a remarkable increase of colon length in β6-KO mice (**Figures 3C, D**). Expectedly, the histology score of β6-KO mice was lower than WT mice (**Figure 3E**). In addition, the pathological evaluation by HE and AB-PAS staining of colitis tissues consistently revealed that β6-KO mice demonstrated milder mucosal damage than WT mice (**Figure 3F**), suggesting lack of integrin β6 attenuated the susceptibility of mice to DSS-induced acute colitis.

### 3.4 Integrin β6 deletion attenuated pro-inflammatory cytokine levels in colitis tissues of mice

**Figure 4:**
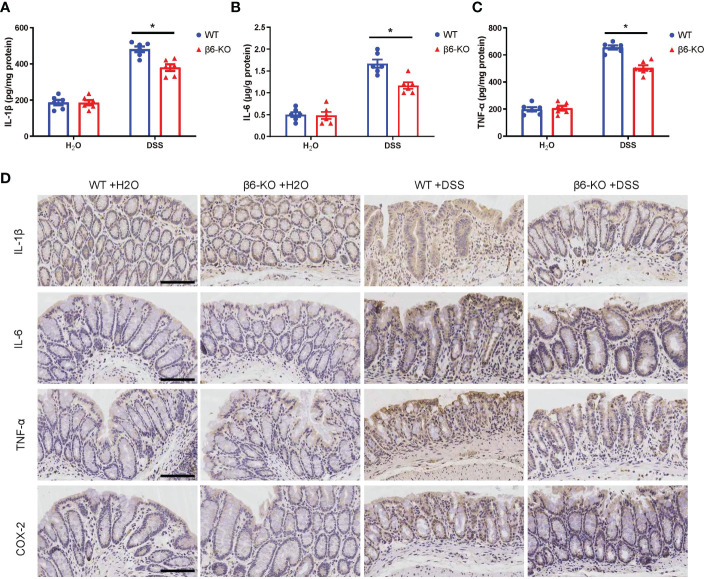
*ITGB6* knockout suppressed the levels of released pro-inflammatory cytokines in colonic tissues of DSS-induced colitis. **(A)** IL-1β, **(B)** IL-6 and **(C)** TNF-α were detected by ELISA assay in the colonic tissues from β6-KO and WT mice in the presence or absence of DSS. **(D)** Representative images of IHC staining of IL-1β, IL-6, TNF-α and COX-2 in the colonic tissues from β6-KO and WT mice in the presence or absence of DSS. Data represented the means ± SEM, ^*^ *p* < 0.05. Scale bar, 100 μm.

Various pro-inflammatory cytokines released by macrophages act as critical regulators of the intestinal inflammatory response in IBD (12). To evaluate the effect of integrin β6 on pro-inflammatory cytokines, ELISA assay was performed on colonic tissues from β6-KO and WT mice after DSS treatment. The levels of IL-1β, TNF-α and IL-6 in the colitis tissues of β6-KO mice were lower than those of WT mice (**Figures 4A–C**). Consistently, IHC staining also confirmed the downregulated expression of IL-1β, IL-6, TNF-α and COX-2 in colitis model of β6-KO mice (**Figure 4D**). These findings implied that lack of integrin β6 could attenuate DSS-induced colitis by suppressing tissue infiltration of pro-inflammatory cytokines.

### 3.5 Loss of integrin β6 attenuated the disruption of tight junctions between colonic epithelial cells in DSS-induced colitis

**Figure 5:**
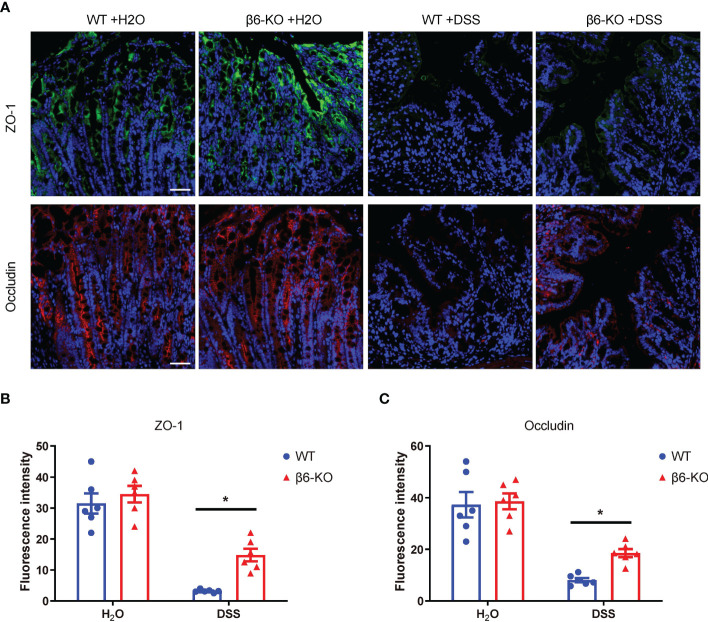
*ITGB6* knockout attenuated the disruption of tight junctions between colonic epithelial cells in DSS-induced colitis. **(A)** Representative images of immunofluorescence staining showing the expression of ZO-1 and occludin in colonic mucosa of WT and β6-KO mice in the presence or absence of DSS treatment. **(B, C)** Analysis of the expression levels of **(B)** ZO-1 and **(C)** occludin in the colonic mucosa of β6-KO and WT mice by immunofluorescence intensity. Data represented the means ± SEM, ^*^ *p* < 0.05. Scale bar, 50 μm. Representative images of IHC staining of IL-1β, IL-6, TNF-α and COX-2 in the colonic tissues from β6-KO and WT mice in the presence or absence of DSS. Data represented the means ± SEM, ^*^ *p* < 0.05. Scale bar, 50 μm.

The basic function of the bowel epithelium is to maintain the structural and functional integrity of the intestinal barrier and to prevent injury to intestinal tissues. One of the typical histopathological features of patients with IBD is the disruption of intestinal barrier. Previous studies have shown that reduced expression and abnormal distribution of tight junction (TJ) proteins contributed to disruption of intestinal barrier function, as observed in patients with active CD and UC (13). Zonula occludens-1 (ZO-1) and occludin are two major TJ proteins, which were hence detected in colonic tissues by immunofluorescence staining. While there was no basic difference regarding the expression of ZO-1 and occludin in the untreated β6-KO and WT mice, loss of integrin β6 could rescue the depressed expression of ZO-1 and occludin in DSS-induced colitis (**Figures 5A–C**). These findings suggested that deletion of integrin β6 elevated the expression of ZO-1 and occludin in colonic tissues, thus attenuating the disruption of colonic epithelial barrier function in DSS-induced colitis.

### 3.6 Integrin β6 deletion altered macrophage polarization in DSS-induced acute colitis

**Figure 6:**
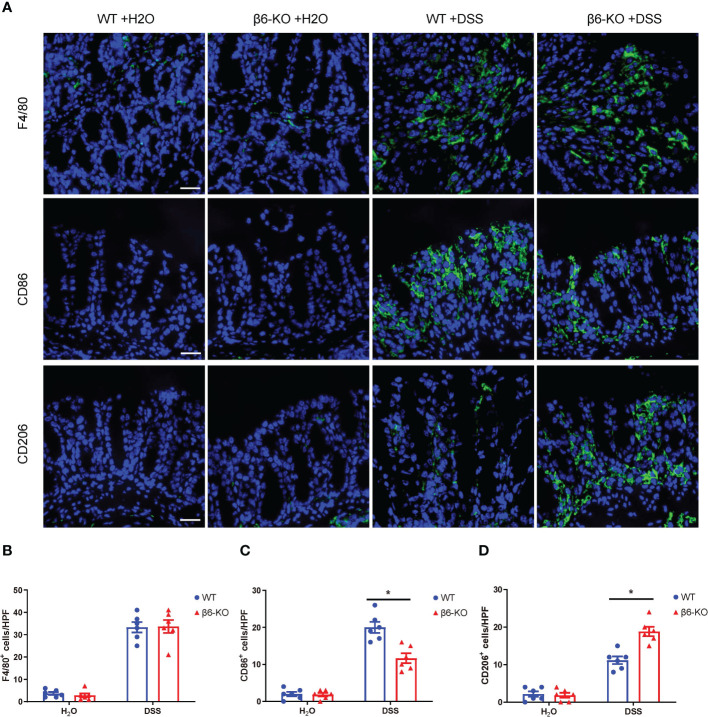
*ITGB6* knockout altered the macrophage polarization in colonic tissue of DSS-induced colitis. **(A)** Immunofluorescence staining images showing the expression of F4/80, CD86 and CD206 in colonic mucosa of WT and β6-KO mice in the presence or absence of DSS treatment. **(B–D)** Quantitative analysis of the number of positively stained cells per high-powered field showing the relative expression level of **(B)** F4/80, **(C)** CD86 and **(D)** CD206 in colonic mucosa of WT and β6-KO mice in the presence or absence of DSS treatment. Data represented the means ± SEM, ^*^ *p* < 0.05. Scale bar, 50 μm.

Prior researches have revealed that dysregulation of intestinal macrophages is involved in the inflammation of IBD. Extensive infiltrating macrophages and cytokines that promote inflammation are uncovered in affected intestinal tissues of patients suffering from colitis. Macrophages, regulated by the microenvironment, are known to be classified into M1 and M2 phenotype according to their function and level of inflammatory cytokine secretion. M1 phenotype macrophages mainly promote the development of inflammation, sterilization and phagocytosis, while M2 phenotype macrophages promote wound healing and tissue repair in a manner of anti-inflammation (14). We here carried out immunofluorescence staining with F4/80 as the marker of macrophages, CD86 as the marker of M1 phenotype, and CD206 as the marker of M2 phenotype to evaluate the effect of integrin β6 deletion on macrophage infiltration and polarization in colitis tissues. There was a large number of macrophages infiltrating the colonic tissue of DSS-induced acute colitis, with the M1 phenotype being predominant (**Figure 6A**). Integrin β6 deletion could remarkably promote the polarization of macrophages into M2 phenotype macrophages, with no effect on the total number of macrophages (**Figures 6A–D**), indicating that lack of integrin β6 could attenuate the inflammation of colitis by promoting macrophage polarization to anti-inflammatory M2 phenotype rather than pro-inflammatory M1 phenotype.

### 3.7 Integrin β6 deficiency inhibited the tumorigenesis and tumor progression of CAC by suppressing macrophage M2 polarization

To investigate the role and mechanism of integrin β6 in the tumorigenesis and tumor progression of CAC, AOM/DSS assay was used to establish CAC model of mice.

**Figure 7:**
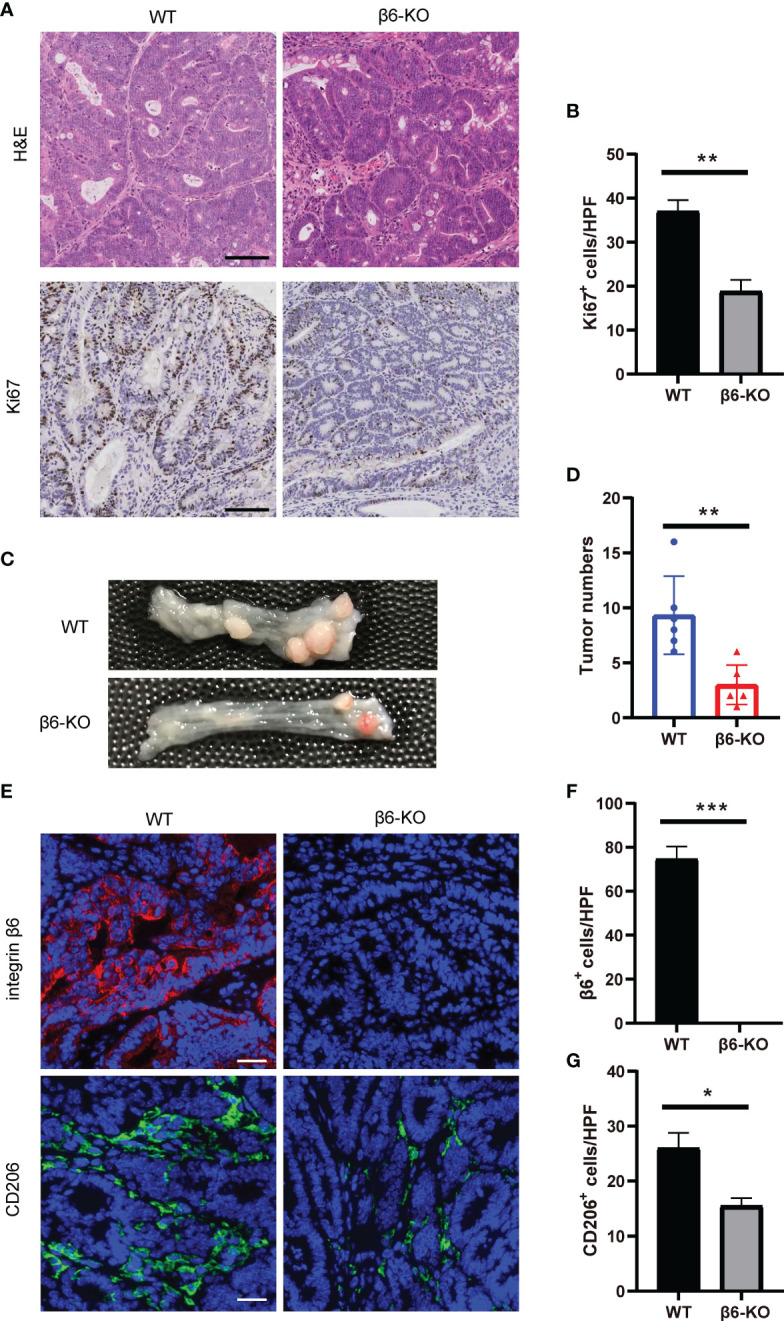
*ITGB6* knockout mice were resistant to CAC by suppressing macrophage M2 polarization. **(A)** Representative images of HE and IHC staining of Ki67 in colonic CAC tissue of integrin β6-KO and WT mice. Scale bar, 100 μm. **(B)** Quantitative analysis of the number of positively stained cells per high-powered field showing expression levels of Ki67. **(C, D)** Representative images of colonic tumors with quantitative analysis of tumor numbers in CAC model of integrin β6-KO and WT mice. **(E)** Representative images of immunofluorescence staining showing the expression of integrin β6 and CD206 in colonic CAC tissue of integrin β6-KO and WT mice. Scale bar, 50 μm. **(F, G)** Quantitative analysis of the count of positively stained cells per high-powered field revealing the expression levels of **(F)** integrin β6 and **(G)** CD206 in colonic CAC tissue of integrin β6-KO and WT mice. Data represented the means ± SEM, ^*^ *p* < 0.05, ^**^ *p* < 0.01, ^***^ *p* < 0.001.

There was a significant decrease in Ki67 staining upon integrin β6 deletion within the tumor tissues (**Figures 7A, B**), indicating reduced number of proliferative cells and tumor growth in CAC caused by *ITGB6* knockout. Furthermore, the number of colon tumors was reduced in integrin β6-KO mice compared with WT mice (**Figures 7C, D**). Therefore, lack of integrin β6 could inhibit tumorigenesis and tumor progression of CAC. Moreover, integrin β6-KO mice demonstrated suppressed expression of CD206 compared with WT mice (**Figures 7E–G**), suggesting reductive infiltration of M2 phenotype macrophages in the tumors led by integrin β6 deletion. Considering that M2 macrophages tend to promote pathological angiogenesis, organ fibrosis and tumor growth (15), we thus speculated that integrin β6 deletion inhibited tumorigenesis by suppressing polarization of macrophages toward M2 phenotype.

## 4 Discussion

As a complex pathogenesis referring to aberrant immune responses contributing to intestinal inflammation, IBD is characterized by being lifelong, recurrent, intractable and carcinogenic. The exact etiology of IBD is yet not well understood, which attracts a large number of researchers dedicated to exploring the pathogenic mechanism of IBD and potential therapeutic targets. Indeed, the migration of leukocytes through the endothelial cell wall is central to the pathogenesis of IBD, and targeted blockade of such process has been shown to be effective and safe (16). Given that anti-integrin agents are able to prevent leukocyte migration through the endothelial cell wall (17), anti-integrin therapy for IBD has already achieved initial promising results in terms of UC. So far, vedolizumab and natalizumab are the only anti-integrin biologics approved for the treatment of IBD (18), which mainly act to limit the inflammatory response by blocking the interaction of integrin α4β7 on the surface of lymphocytes with the addressin on the intestinal endothelium, thereby blocking the migration of leukocytes to the intestinal epithelium (19). However, since integrin α4β7 is mainly expressed in circulating lymphocytes, anti-integrin agents may, more or less, cause systemic side effects such as progressive multifocal leukoencephalopathy, infections and gastrointestinal events (20, 21). Integrin αvβ6 is expected to overcome this problem because it is an epithelium-restricted cell surface receptor. In the present study, we demonstrated that integrin β6 was highly expressed in inflammatory tissues and tumors in both patients and experimental mouse models, with positive correlation with the severity of inflammation.

Integrin αvβ6 is barely expressed in normal adult tissues, but its expression becomes dramatically elevated during tissue repair and malignancy (22). However, few studies have investigated the role of integrin αvβ6 in IBD. Rydell et al. found that the level of serum IgG anti-integrin αvβ6 autoantibodies was obviously increased in patients with UC (23), consistent with our finding that integrin αvβ6 was abundantly expressed in the intestinal epithelium of UC. We also found integrin αvβ6 expression was related with the severity of inflammation, in accordance with the previous report which through *ITGB6* transgenic mouse model found that integrin β6 maintained the colon hyperresponsive to inflammatory factors and ultimately promoted the development of IBD (10). What’s more, we observed that the expression of integrin β6 was elevated in other inflammatory organ tissues such as gastritis, pancreatitis and pneumonitis, which verified that integrin β6 could be *de novo* synthesized in inflammatory events with tissue remodeling. In the present study, integrin β6 deficient mice were generated, which showed that *ITGB6* knockout significantly reduced susceptibility to DSS-induced colitis. It is well known that IBD exhibits an overactivation of the inflammatory process, with one manifestation by the production of pro-inflammatory cytokines. Accordingly, we found that integrin β6 deficiency remarkably decreased the release of pro-inflammatory cytokines including TNF-α, IL-6 and IL-1β in the intestinal tissues of colitis.

Keeping mucosal epithelium intact is essential to avoid intestinal tissue damage and prevent inflammation. Abnormal expression of TJ proteins in patients with active IBD leads to disruption of barrier function. In addition, TJ dysregulation may be associated with activation of cytokines with different transcriptional and post-transcriptional mechanisms or induced intestinal inflammation (24). However, there is a large gap in our knowledge regarding the molecular mechanisms regulating TJ barrier function during disease manifestation. Integrin αvβ6 is mainly restricted to epithelial cells where it plays a role in maintaining epithelial barrier function (25). In the present study, knockout of integrin β6 promoted the expression of TJ proteins ZO-1 and occludin in mice with DSS-induced acute colitis, implying the attenuated disruption of TJ between colonic epithelial cells probably due to suppressed release of pro-inflammatory cytokines by integrin β6 deletion.

Macrophages are thought to be a major factor in keeping the intestinal environment stable through modulation of inflammation. Dysregulation of intestinal macrophages is reported to be responsible for chronic inflammation of IBD (26). Imbalanced polarization and ratio of M1/M2 macrophages to a large degree determine the response of tissue to injury or inflammation. Normally, M1 phenotype macrophages evoke a proper pro-inflammatory response that facilitates the eradication of intracellular infections to maintain a stable intestinal environment (27). However, dysregulation of M1 phenotype macrophages due to imbalanced polarization leads to a high production of pro-inflammatory cytokines, rendering the intestine susceptible to infection, inflammation and tumor lesions (28). Elevated filtration of M1 phenotype macrophages and level of pro-inflammatory cytokines are measured in the intestines of patients suffering from chronic colitis, while decreased number of M2 phenotype macrophages and level of anti-inflammatory cytokines were observed (29). In addition, in the affected intestine with UC, macrophages deliver antigens and contribute to the progression of UC to CAC *via* engulfing activities and production of chemokines and inflammatory cytokines (30). Thus, macrophage polarization may be specifically associated with the development of IBD.

Pro-inflammatory M1 phenotype macrophages can directly contribute to the damage of the intestinal epithelial barrier, while pro-angiogenic and pro-”wound healing” M2 phenotype macrophages may act antagonistically and modulate the inflammatory response (31, 32). The occurrence and development of CAC is associated with a pronounced filtration of macrophage, especially M2 phenotype macrophages which have been demonstrated to be associated with tumor growth, invasion, metastasis, and poor prognosis (33, 34). Our study found that knockout of integrin β6 could inhibit the polarization of macrophages to M1 phenotype but promote macrophages polarization to M2 phenotype, which attenuated the degree of inflammation in mice suffering from DSS-induced colitis. On the other hand, integrin β6 knockout could inhibit tumorigenesis and development by suppressing macrophage polarization toward M2 phenotype in CAC mice model. The seemingly contradictory effects of integrin β6 deletion could be context specific through different mechanisms on polarization to M2 phenotype macrophage in inflammation and tumorigenesis. Integrin β6 activates TGF-β, which inhibits nitric oxide synthesis and stimulates arginase activity, thus appearing to regulate the balance of M1/M2 phenotype macrophages (35). However, the exact mechanism needs to be further explored. Nonetheless, strategies regulating macrophage infiltration and polarization could be a promising choice for controlling inflammation and suppressing colitis-associated carcinogenesis in patients with colitis (36). The main drawback of current strategies inactivating macrophages is the systemic and indiscriminate targeting of macrophages, which leaves the immune response compromised at a disadvantage in fighting external attacks. Therefore, it is necessary to find IBD-related specific targets to replace the strategy indiscriminately targeting macrophages in order to achieve disease remission while reducing systemic side effects. The present study suggests that targeting integrin β6 may potentially be an alternative strategy to target macrophage polarization in the treatment of IBD.

## 5 Conclusion

Despite the partial success of biologic and small molecule agents in the management of IBD, there is still a proportion of patients who fail to response or develop drug resistance. Furthermore, with the development of available therapies, the demand for minimizing side effects is increasing. From this perspective, our present study is of significance, showing that integrin β6 is a potential target for the treatment of IBD by overcoming the shortcomings of current anti-integrin therapies and macrophage clearance/inactivation strategies, since integrin β6 is highly expressed only in the intestinal epithelium of IBD and promotes the development of IBD by affecting macrophage polarization.

## Data availability statement

The original contributions presented in the study are included in the article/supplementary material. Further inquiries can be directed to the corresponding author.

## Ethics statement

The studies involving human participants were reviewed and approved by the Ethics Committee of The First Affiliated Hospital of Xi’an Jiaotong University. The patients/participants provided their written informed consent to participate in this study. The animal study was reviewed and approved by the Ethics Committee of The First Affiliated Hospital of Xi’an Jiaotong University. Written informed consent was obtained from the individual(s) for the publication of any potentially identifiable images or data included in this article.

## Author contributions

QS, ZL and FL designed and conducted the experiments, and drafted the manuscript. LM, DX and CL assisted in the study design and data analysis. CY and WH performed the data collection. YD consulted the literature and help revision. FL designed the study, supervised the project. FL edited the manuscript. All authors contributed to the article and approved the submitted version.
